# Olfactory Neuroblastoma Causing Cushing’s Syndrome Due to the Ectopic Adrenocorticotropic Hormone (ACTH) Secretion: A Case Report

**DOI:** 10.7759/cureus.56434

**Published:** 2024-03-19

**Authors:** Abdulrahman A Alsarari, Anas A Abdulkader, Waqar A Farooqi, Salwa K Al-Shibani, Tarig S Al Khuwaitir

**Affiliations:** 1 Medicine and Surgery, Al Maarefa University, Riyadh, SAU; 2 Internal Medicine, Al Maarefa University, Riyadh, SAU; 3 Anatomic Pathology, Ad-Diriyah Hospital, Ministry of Health, Riyadh, SAU; 4 Internal Medicine, King Saud Medical City, Riyadh, SAU

**Keywords:** case report, hypokalemia, ectopic acth syndrome (eas), cushing’s syndrome, olfactory neuroblastoma

## Abstract

Cushing’s syndrome is a constellation of features occurring due to high blood cortisol levels. We report a case of a 47-year-old male with a history of recurrent olfactory neuroblastoma (ONB). He presented with bilateral lower limb weakness and anosmia and was found to have Cushing’s syndrome due to high adrenocorticotropic hormone (ACTH) levels from an ectopic source, ONB in this case. Serum cortisol and ACTH levels declined after tumor removal.

## Introduction

Olfactory neuroblastoma (ONB), or esthesioneuroblastoma, is a rare malignancy arising from neuroepithelium in the upper nasal cavity. It represents approximately 2% of all nasal passage tumors, with an incidence of approximately 0.4 per 2.5 million individuals [1]. ONB shares similar histological features with small round blue cell neoplasms of the nose. Ectopic hormone secretion is a very rare feature associated with these tumors. Five-year overall survival is reported to be between 60% and 80% [2,3]. The age distribution is either in the fifth to sixth decade of life [4,5], or in the second and sixth decades [6].

Features of Cushing’s syndrome (moon face, buffalo hump, central obesity hypertension, fragile skin, easy bruising, fatigue, muscle weakness) are due to high blood cortisol levels [7]. It can be either primary (cortisol-secreting adrenal tumor), secondary (adrenocorticotropic hormone (ACTH)-secreting pituitary tumor, also called Cushing disease), or ectopic ACTH secretion (from a non-pituitary source). All three types share similar features [8].

Ectopic ACTH syndrome (EAS) is due to an extra pituitary tumor, producing ACTH. It accounts for 12-17% of Cushing's syndrome cases [9]. Most cases of EAS-producing tumors are in the lungs, mediastinum, neuroendocrine tumors of the gastrointestinal tract, and pheochromocytomas [9]. Ectopic ACTH secretion from an ONB is very rare. As of 2015, only 18 cases were reported in the literature [10]. Here, we report such a case.

## Case presentation

Our patient is a 47-year-old Bangladeshi male, with a history of recurrent ONB that was resected twice in the past (transsphenoidal resection in 2016 and 2019) with adjuvant radiotherapy, no chemotherapy was given. He also had diabetes mellitus type 1 (poorly controlled) and hypertension. He presented with bilateral lower limb weakness, anosmia, decreased oral intake, loss of taste for one week, and bilateral submandibular swelling that increased in size gradually over the past two years. There was no history of fever, cough, abdominal pain, or exposure to sick contacts. The patient reported past episodes of similar symptoms, but details are unclear. The patient's family history is positive for diabetes mellitus type 1 in both parents. Lab tests in the emergency department showed hypokalemia and hyperglycemia as detailed in Table 1. He was admitted for further workup of the above complaints.

**Table 1:** Results of blood test at the time of hospitalization. Hypokalemia and high values of adrenocorticotropic hormone and cortisol were confirmed.

Test	Patient Results	Reference Range	Unit	Status
Hemoglobin	14.7	13-17	g/dL	Normal
White blood cell (WBC)	17.9	4-10	10*9/L	High
Neutrophils	15.89	2-7	10*9/L	High
Lymphocytes	1.07	1-3	10*9/L	Normal
Sodium	141	136-145	mmol/L	Normal
Potassium	2.49	3.5-5.1	mmol/L	Low (Panic)
Chloride	95	98-107	mmol/L	Low
Glucose	6.52	4.11-5.89	mmol/L	Elevated
C-reactive protein (CRP)	0.64	Less than 5	mg/L	Normal
Erythrocyte sedimentation rate (ESR)	2	0-30	mm/h	Normal
Creatinine	73	62-106	µmol/L	Normal
Uric acid	197	202.3-416.5	µmol/L	Normal
Alanine aminotransferase (ALT)	33.2	0-41	U/L	Normal
Aspartate aminotransferase (AST)	18.6	0-40	U/L	Normal
International Normalised Ratio (INR)	1.21	0.8-1.2	sec	High
Prothrombin time (PT)	15.7	12.3-14.7	sec	High
Lactate dehydrogenase (LDH)	491	135-225	U/L	High
Thyroid-stimulating hormone (TSH)	0.222	0.27-4.20	mIU/L	Low
Adrenocorticotropic hormone (ACTH)	106	≤50	ng/L	Elevated
Cortisol (after dexamethasone suppression)	1750	Morning hours (6-10 am): 172-497 nmol, Afternoon hours (4-8 pm): 74.1-286 nmol	nmol/L	Elevated (failure of suppression)
24-hour urine cortisol (after dexamethasone suppression)	5959.1	<120 nmol/24 hrs	nmol/24hr	Elevated (failure of suppression)

On examination, the patient's vital signs were as follows: blood pressure was 154/77 mmHg, heart rate of 60 beats per minute, respiratory rate was 18 breaths per minute, oxygen saturation of 98% on room air, and a temperature of 36.7°C. The patient had a typical Cushingoid appearance with a moon face, buffalo hump, purple striae on the abdomen, central obesity, and hyperpigmentation of the skin. Submandibular lymph nodes were enlarged bilaterally. The examination of the submandibular lymph nodes showed a firm, fixed mass extending from the angle of the mandible to the submental space on the left side. Neurological examination showed weakness in both legs bilaterally (strength 3/5) and anosmia (checked by orthonasal smell test). The rest of the neurological exam was normal.

Laboratory findings revealed (in Table 1) a marked hypokalemia of 2.49 mmol/L and hyperglycemia of 6.52 mmol/L. The serum cortisol level was elevated at 1587 nmol/L. Serum ACTH levels were raised at 106 ng/L (normal value ≤50 ng/L). Moreover, the high-dose dexamethasone suppression test failed to lower the serum ACTH levels and serum and urine cortisol. Serum cortisol level after the suppression test was 1750 nmol/L, while 24-hour urine cortisol after the test was 5959.1 nmol/24hr. Serum ACTH levels after the test also remained high at 100mg/L. This indicated failure of ACTH suppression by high-dose dexamethasone, which points towards ectopic ACTH production. Other blood tests (complete blood count, liver function tests) were insignificant.

**Figure 1:**
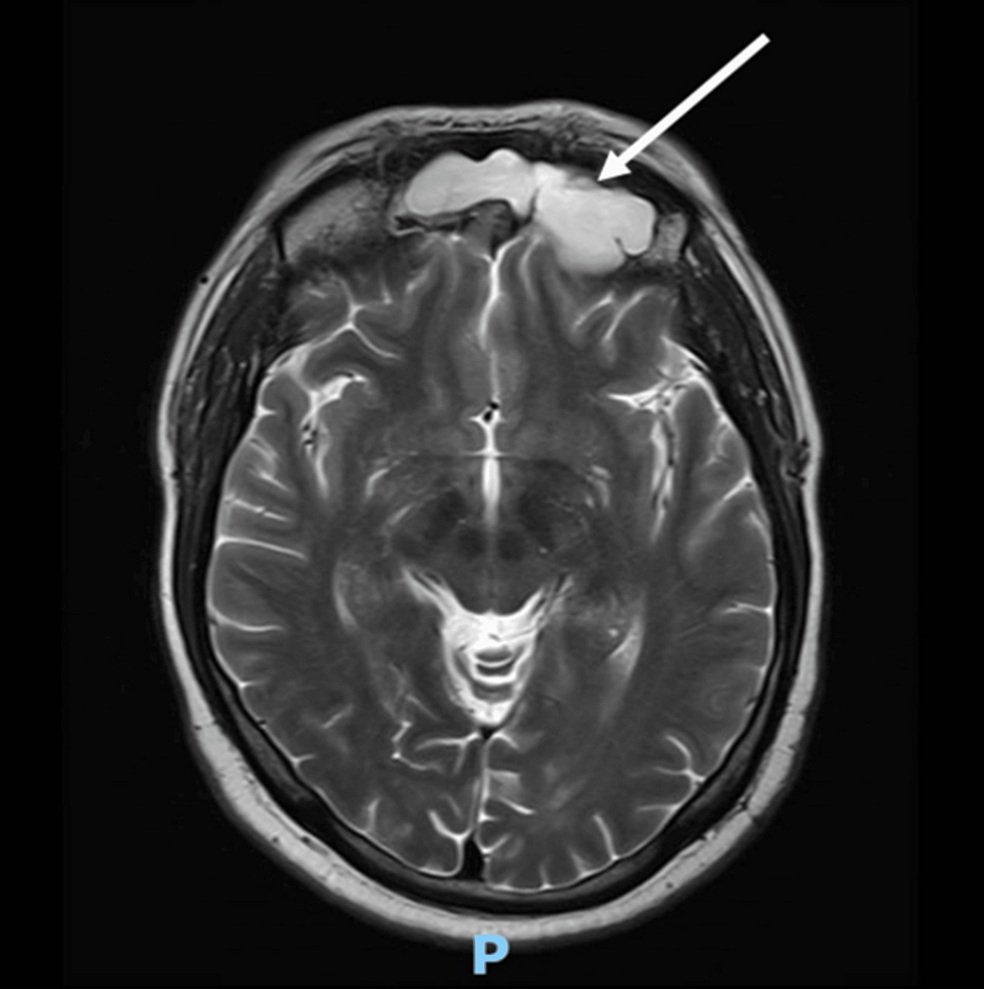
A brain MRI (T1-weighted; without contrast; sagittal plane) shows a soft tissue lesion located in the midline olfactory groove area. Dural surface with extension into anterior frontal dura. MRI: Magnetic resonance imaging

**Figure 2:**
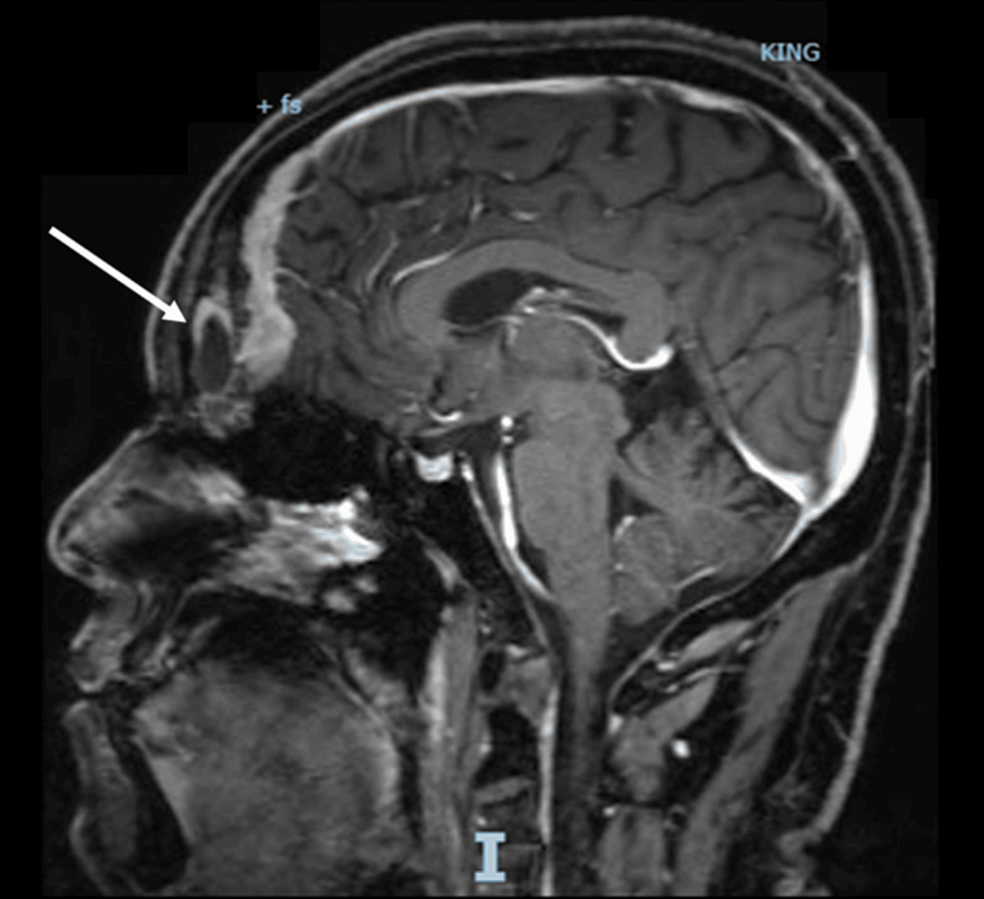
A brain MRI (T2-weighted; without contrast; axial plane) shows a soft tissue lesion located in the midline olfactory groove area. MRI: Magnetic resonance imaging

A computed tomography scan with contrast (CT scan) of the chest, abdomen, and pelvis, with a special focus on the adrenals, was negative for any malignancy or masses. CT scan of the neck showed bilaterally enlarged submandibular lymph nodes and an enlarged right lobe of the thyroid with nodules. Fine needle aspiration (FNA) of the thyroid nodules revealed a benign nature. Magnetic resonance imaging (MRI) of the brain showed a contrast-enhancing soft tissue lesion (18x18x10mm) in the midline olfactory groove area with extension into the frontal dura and superior sagittal sinus, suggesting recurrence of the previous ONB. There was evidence of previous surgery also. The pituitary gland was normal (Figures 1-2).

**Figure 3:**
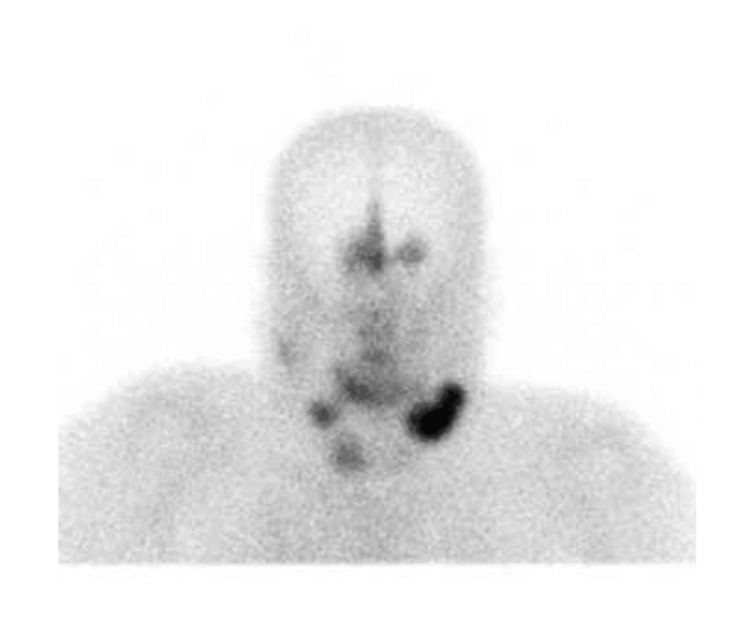
Whole-body octreotide scan (15 mCi 99mTc-Octreotide IV) demonstrates three focal abnormal uptakes: the largest (5.2 x 2.4 cm) in the left submandibular region, and two smaller ones on the right, suggestive of lymph node uptake. Additional abnormal uptake was seen along the midline of the olfactory groove region with bilateral extension. No other significant abnormal uptake was identified.

Octreotide scintigraphy showed three focal abnormal uptakes in the submandibular cervical nodes. Additionally, there was a moderate abnormal uptake at the midline olfactory groove with bilateral extension (Figure 3).

**Figure 4:**
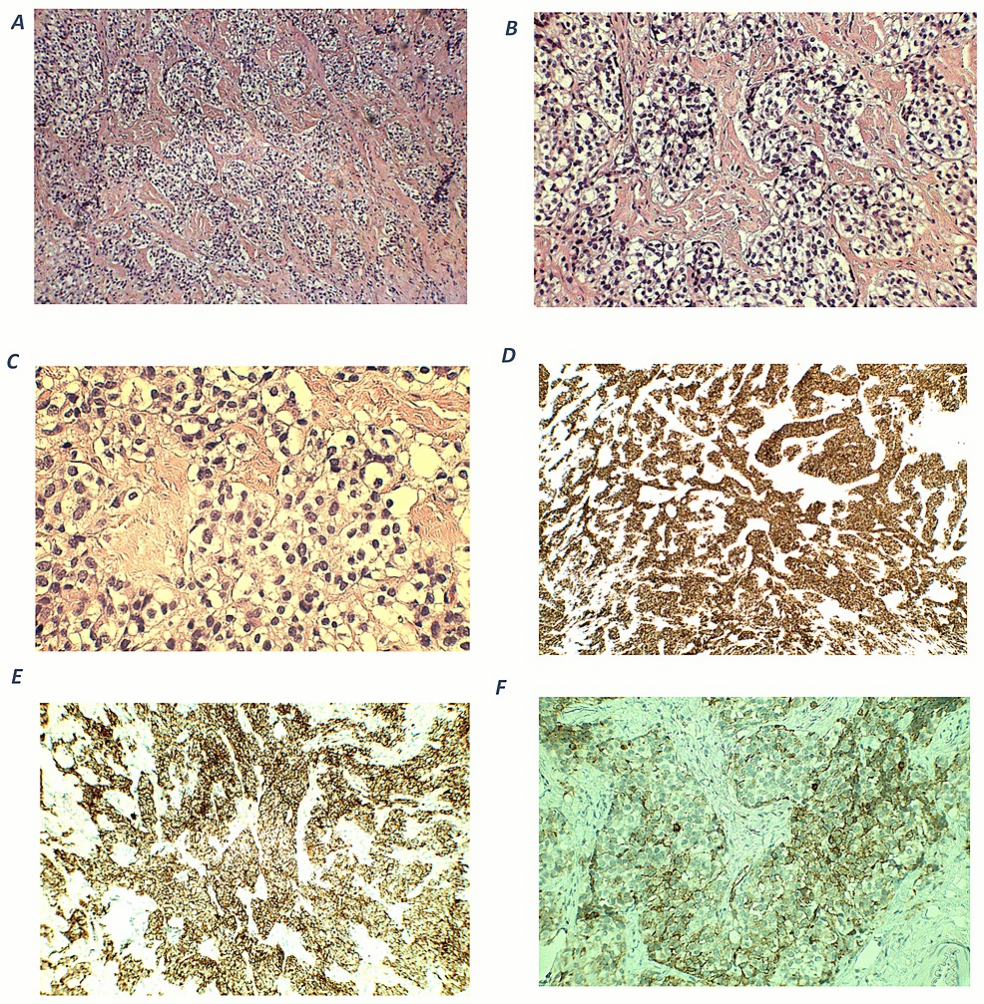
Histopathological and immunohistochemical findings of olfactory neuroblastoma. A (100x magnification) and B (200x magnification) - hematoxylin and eosin (H-E) staining shows cellular nests of round blue cells separated by hyalinized stroma. C (400x magnification) - nuclei show mild to moderate pleomorphism with fine chromatin. D (100x magnification) - an immunohistochemical stain for synaptophysin shows diffuse, strong cytoplasmic positivity within tumor cells. E (200x magnification) - tumor cells are positive for chromogranin. F (400x magnification) - ACTH cytoplasmic expression in tumor cells. ACTH: adrenocorticotropic hormone

On microscopic examination, an excisional biopsy after the transcranial resection surgery of the frontal skull base tumor showed nests and lobules of round to oval cells with clear cytoplasm, separated by vascular and hyalinized fibrous stroma (Figures 4A-4B). Tumor cells show mild to moderate nuclear pleomorphism, and fine chromatin (Figure 4C). A fibrillary neural matrix is also present. Some mitotic figures can be seen. Immunohistochemical stains revealed positive staining for synaptophysin (Figure 4D) and chromogranin (Figure 4E). Stains for CK (AE1/AE3), CD45, Desmin, and Myogenin are negative. Immunostaining for ACTH was focally positive (Figure 4F), while the specimen of the cervical lymph nodes showed the same staining, indicating metastases. The cytomorphologic and immunophenotypic features observed are consistent with a Hyams grade II ONB, with ectopic ACTH production.

For his resistant hypokalemia, he had to be given intravenous (IV) and oral potassium chloride (KCL) repeatedly. The patient underwent transcranial resection of the frontal skull base tumor. The patient received cefazolin for seven days, and hydrocortisone for four days. After transcranial resection, his cortisol level decreased to 700 nmol/L. Furthermore, ACTH dropped, and serum potassium also normalized. Subsequently, the patient was transferred to the intensive care unit (ICU) for meticulous monitoring and continued care. In the ICU, the patient developed one episode of a generalized tonic-clonic seizure, which aborted spontaneously, and the patient received phenytoin and levetiracetam to prevent other episodes. A right-sided internal jugular vein and left transverse sinus thrombosis were also developed and treated with enoxaparin sodium. Following surgery, his low potassium levels improved, resulting in an improvement in his limb weakness. His other symptoms also gradually improved after surgery. Three weeks following the primary tumor resection, he underwent bilateral neck dissection with right hemithyroidectomy, for removal of the metastases. The patient opted out of chemotherapy and planned for an international transfer to his home country for further management. Other treatments that he received during hospitalization were ceftriaxone, azithromycin, and Augmentin®. Insulin was used to manage his diabetes, perindopril to regulate his blood pressure, and spironolactone to increase potassium retention. Omeprazole was administered to prevent GI bleeding and heartburn/gastroesophageal reflux disease relief after discharge.

## Discussion

ONB was first described in 1924, and it is a rare neuroectodermal tumor that accounts for 2% of tumors affecting the nasal cavity [11]. Even though ONB has a good survival rate, long-term follow-up is necessary due to the disease's high recurrence rate [2]. ONB recurrence has been approximated to range between 30% and 60% after successful treatment of the primary tumor [12]. Recurrent disease is usually locoregional and tends to have a long interval to relapse with a mean of six years [12]. The first reported case of ectopic ACTH syndrome caused by ONB was in 1987 by M Reznik et al., who reported a 48-year-old woman with ONB who developed a Cushing-like syndrome 28 months before her death [13].

The occurrence of Cushing’s syndrome due to ectopic ACTH can occur either in the initial tumor or even years later during its course or after recurrence [3,6,9,14]. Similar to the case of Abe et al. [3], our patient also presented with muscle weakness due to hypokalemia, which is a feature of Cushing’s syndrome. Hypokalemia is present at diagnosis in 64% to 86% of cases of EAS and is resistant to treatment [9,14], as seen in our case. In our patient, the exact time of development of Cushing’s syndrome could not be ascertained due to the non-availability of previous records. However, according to the patient, he started developing abdominal obesity, pigmentation, and buffalo hump in 2021 about two years after his second surgery for ONB.

The distinction between pituitary ACTH and ectopic ACTH involves utilizing CT/MRI of the pituitary, corticotropin-releasing hormone (CRH) stimulation test with petrosal sinus blood sampling, high dose dexamethasone suppression test, and checking serum K+ (more commonly low in ectopic ACTH) [2,15,16]. In our case, a CRH stimulation test was not available but CT/MRI brain, dexamethasone test, low serum potassium, plus the postoperative fall in cortisol levels, all pointed towards an ectopic ACTH source.

## Conclusions

In conclusion, this case highlights the rare association between ONB and ectopic ACTH syndrome, which developed after tumor recurrence. The patient's unique presentation of bilateral lower limb weakness and hypokalemia can cause diagnostic challenges, emphasizing the need for comprehensive diagnostic measures. Surgical intervention proved crucial, with postoperative cortisol values becoming normal, highlighting the efficacy of this approach. The occurrence of ectopic ACTH production in ONB patients, although very rare, is emphasized, so that healthcare professionals who deal with these tumors are aware of this complication. This report contributes valuable insights shedding light on the unique ONB manifestation causing ectopic ACTH syndrome. The ongoing monitoring of the patient's clinical features will further enrich the understanding of the course of this uncommon phenomenon in the medical literature.
